# Crystal structure of racemic *cis*-2-amino-1,2-di­phenyl­ethanol (ADE)

**DOI:** 10.1107/S2056989015022318

**Published:** 2015-11-28

**Authors:** Isao Fujii

**Affiliations:** a School of Science, Tokai University, 4-1-1 Kitakaname, Hiratuka, Kanagawa 259-1292, Japan

**Keywords:** crystal structure, optical resolving agent, 2_1_-helical columnar structure, inter­molecular hydrogen bonding, C—H⋯π and N—H⋯π inter­actions

## Abstract

In the title racemic compound, C_14_H_15_NO, the hy­droxy and amino groups form a bent tweezer-like motif towards the phenyl groups. In the crystal, enanti­omers aggregate with each other and are linked by O—H⋯N hydrogen bonds, forming chiral 2_1_-helical columnar structures from *C*(5) chains along the *b*-axis direction. Left- and right-handed 2_1_ helices are formed from (1*S*,2*R*)-2-amino-1,2-di­phenyl­ethanol and (1*R*,2*S*)-2-amino-1,2-di­phenyl­ethanol, respectively.

## Abstract 2

In the crystal of the title racemic compound, enanti­omers aggregate with each other and are linked by O—H⋯N hydrogen bonds to form chiral 2_1_-helical columnar structures from *C*(5) chains along the *b*-axis direction.

## Chemical context   

The production of chiral compounds has great importance in the pharmaceutical industry, and diastereomer salt separation is still widely applied in the process. An optical resolving agent, chiral 2-amino-1,2-di­phenyl­ethanol (ADE) (Read & Steele, 1927 ▸), has been widely tried and used in diastereomer salt separation methods; for example, chiral discrimination of 2-aryl­alkanoic acids by (1*R*,2*S*)-ADE (*cis*-isomer) (Kinbara *et al.*, 1998 ▸). The ADE mol­ecule with two adjacent stereogenic centers exists as diastereoisomers (and more, enanti­omers of *cis-* and *trans-*forms), and can be purchased without difficulty. It was considered that *cis-* and *trans-*ADE have different properties and play different roles in diastereomer salt separations. In fact, co-crystal structures with *cis-*ADE enanti­omers have been found in previous reports. The racemic structure of *trans-*ADE has been reported (Bari *et al.*, 2012 ▸), but that of *cis-*ADE has not. The crystal structure of racemic *cis-*ADE is reported on herein.
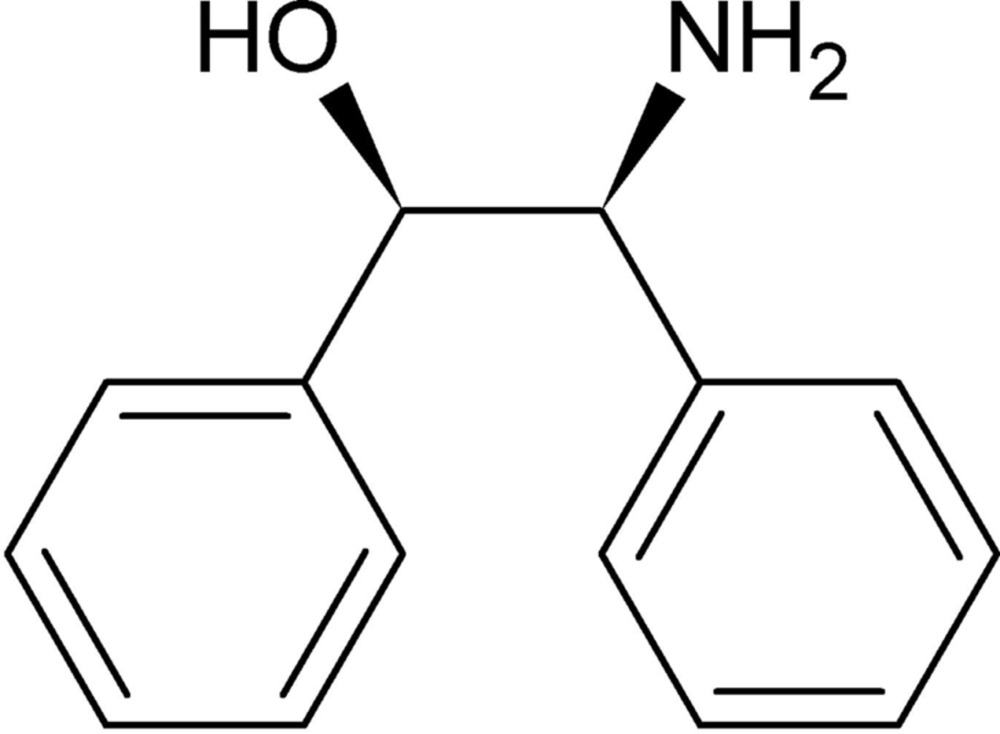


## Structural commentary   

**Figure 1:**
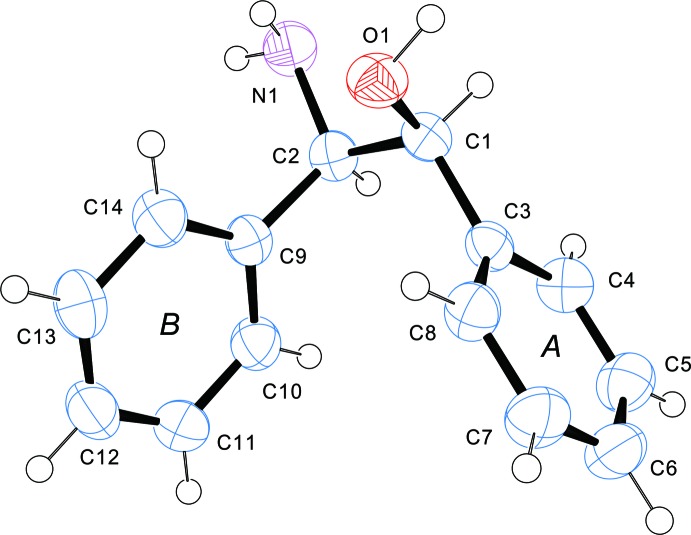
A view of the mol­ecular structure of *cis-(1S,2R)*-ADE, with atom and ring labelling. Displacement ellipsoids are drawn at the 50% probability level.

In the title compound (*cis*-ADE), Fig. 1 ▸, the hy­droxy and amino groups form a tweezer-like motif. Selected geometrical parameters are given in Table 1 ▸. The dihedral angle between the phenyl rings is 50.29 (6)° and the torsion angle O1—C1—C2—N1 is 59.72 (11)°. These values are similar to those observed for *trans*-ADE (Bari *et al.*, 2012 ▸), *viz*. 48.05 (5) and 54.01 (10)°, respectively. However, in *cis*-ADE the hydroxyl group against the opposed phenyl ring adopts a *gauche* conformation [O1—C1—C2—C9 = −67.39 (11)°] compared to a *trans* conformation in *trans*-ADE. Thus a tweezer-like motif bent against the phenyl groups is seen in *cis*-ADE *versus* a projected motif in *trans*-ADE. The arrangements are similar to those found in the diastereomer salts with *cis*-enanti­omers, except for (1*R*,2*S*)-2-ammonio-1,2-di­phenyl­ethanol (Imai *et al.*, 2008 ▸).

## Supra­molecular features   

**Figure 2:**
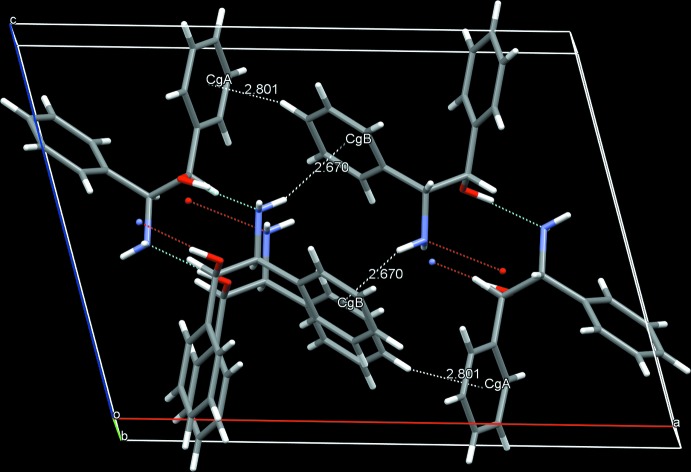
A partial view of the crystal packing of the title compound. Dashed lines indicate the hydrogen bonds, and C—H⋯π and N—H⋯π inter­actions (see Table 2 ▸).

In the crystal, enanti­omers aggregate separately and are linked by O1—H13⋯N1 = [2.7977 (16) Å] hydrogen bonds, forming chiral 2_1_-helical columnar structures from *C*(5) chains along the *b*-axis direction (Table 2 ▸ and Fig. 2 ▸): Left- and right-handed 2_1_ helices are formed from (1*S*, 2*R*)-ADE and (1*R*, 2*S*)-ADE, respectively. The hydro­phobic columnar structures surrounded by phenyl groups are consolidated by the C—H⋯π and N—H⋯π inter­actions, forming slabs parallel to the *ab* plane (Table 2 ▸ and Fig. 2 ▸). This is in contrast to the columnar structure stacking of racemic *R* ^2^_*2*_(10) ring dimers from the O—H⋯N hydrogen bonds observed in the crystal structure of *trans-*ADE (Bari *et al.*, 2012 ▸).

## Synthesis and crystallization   

*cis*-Enanti­omers of 2-amino-1,2-di­phenyl­ethanol (ADE) were purchased from Sigma–Aldrich Co. Ltd. Equivalent weights were mixed in a bottle. Plate-like colourless crystals of the title racemic compound were obtained by vapour-phase diffusion of an aqueous ethanol solution at 297 K.

## Refinement   

Crystal data, data collection and structure refinement details are summarized in Table 3 ▸. All H atoms were located in difference Fourier maps. The NH_2_ and OH H atoms were freely refined. The C-bound H atoms were included in calculated positions and treated as riding atoms: C—H = 0.93–0.98 Å with *U*_iso_(H) = 1.2*U*_eq_(C).

## Supplementary Material

Crystal structure: contains datablock(s) global, I. DOI: 10.1107/S2056989015022318/su5243sup1.cif

Structure factors: contains datablock(s) I. DOI: 10.1107/S2056989015022318/su5243Isup2.hkl

CCDC reference: 1438134

Additional supporting information:  crystallographic information; 3D view; checkCIF report

## Figures and Tables

**Table 1:** Selected geometric parameters (Å, °)

O1—C1	1.4213 (14)	N1—C2	1.4732 (15)
			
O1—C1—C3	112.57 (9)	N1—C2—C9	115.19 (9)
O1—C1—C2	107.90 (9)	N1—C2—C1	106.72 (9)
			
O1—C1—C2—N1	59.72 (11)	C3—C1—C2—N1	−175.47 (9)
O1—C1—C2—C9	−67.39 (11)	C3—C1—C2—C9	57.42 (12)

**Table 2:** Hydrogen-bond geometry (Å, °) *CgA* and *CgB* are the centroids of rings C3–C8 and C9–C14, respectively.

*D*—H⋯*A*	*D*—H	H⋯*A*	*D*⋯*A*	*D*—H⋯*A*
O1—H13⋯N1^i^	0.95 (2)	1.86 (2)	2.7977 (16)	173.1 (16)
N1—H15⋯*CgB* ^ii^	0.88 (2)	2.670 (19)	3.5125 (14)	160.3 (15)
C12—H10⋯*CgA* ^iii^	0.93	2.80	3.6780 (17)	158

Symmetry codes: (i) 
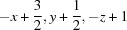
; (ii) 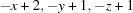; (iii) 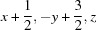.

**Table 3:** Experimental details

Crystal data	
Chemical formula	C_14_H_15_NO
*M*_r_	213.27
Crystal system, space group	Monoclinic, *P*2_1_/*a*
Temperature (K)	297
*a*, *b*, *c* (Å)	16.7752 (17), 5.7573 (10), 12.2887 (13)
β (°)	105.680 (7)
*V* (Å^3^)	1142.7 (3)
*Z*	4
Radiation type	Cu *K*α
μ (mm^−1^)	0.61
Crystal size (mm)	0.30 × 0.30 × 0.20

Data collection	
Diffractometer	Entaf–Nonius CAD-4
Absorption correction	ψ scan (North *et al.*, 1968 ▸)
*T*_min_, *T*_max_	0.83, 0.90
No. of measured, independent and observed [*I* > 2σ(*I*)] reflections	2442, 2354, 2058
*R*_int_	0.019
(sin θ/λ)_max_ (Å^−1^)	0.626

Refinement	
*R*[*F* ^2^ > 2σ(*F* ^2^)], *wR*(*F* ^2^), *S*	0.037, 0.105, 1.03
No. of reflections	2354
No. of parameters	158
H-atom treatment	H atoms treated by a mixture of independent and constrained refinement
Δρ_max_, Δρ_min_ (e Å^−3^)	0.22, −0.18

Computer programs: *CAD-4 Software* (Enraf–Nonius, 1989 ▸), *XCAD4* (Harms & Wocadlo, 1995 ▸), *SHELXS97* (Sheldrick, 2008 ▸), *SHELXL2014/7* (Sheldrick, 2015 ▸), *ORTEP-3 for Windows* and *WinGX* (Farrugia, 2012 ▸), *Mercury* (Macrae *et al.*, 2008 ▸), *PLATON* (Spek, 2009 ▸) and *publCIF* (Westrip, 2010 ▸).

## supplementary crystallographic information

### Crystal data

**Table:**

C_14_H_15_NO	*F*(000) = 456
*M**_r_* = 213.27	*D*_x_ = 1.240 Mg m^−^^3^
Monoclinic, *P*2_1_/*a*	Cu *K*α radiation, λ = 1.54178 Å
Hall symbol: -P 2yab	Cell parameters from 25 reflections
*a* = 16.7752 (17) Å	θ = 20–25°
*b* = 5.7573 (10) Å	µ = 0.61 mm^−^^1^
*c* = 12.2887 (13) Å	*T* = 297 K
β = 105.680 (7)°	Plate, colourless
*V* = 1142.7 (3) Å^3^	0.30 × 0.30 × 0.20 mm
*Z* = 4	

### Data collection

**Table:**

Entaf–Nonius CAD-4 diffractometer	2058 reflections with *I* > 2σ(*I*)
Radiation source: tube sealed	*R*_int_ = 0.019
Graphite monochromator	θ_max_ = 74.9°, θ_min_ = 3.7°
2θ–ω scan	*h* = −21→0
Absorption correction: ψ scan (North *et al.*, 1968)	*k* = −7→0
*T*_min_ = 0.83, *T*_max_ = 0.90	*l* = −14→15
2442 measured reflections	3 standard reflections every 300 reflections
2354 independent reflections	intensity decay: none

### Refinement

**Table:**

Refinement on *F*^2^	H atoms treated by a mixture of independent and constrained refinement
Least-squares matrix: full	W = 1/[Σ^2^(*F*O^2^) + (0.0588*P*)^2^ + 0.2117*P*] WHERE *P* = (*F*O^2^ + 2*F*C^2^)/3
*R*[*F*^2^ > 2σ(*F*^2^)] = 0.037	(Δ/σ)_max_ < 0.001
*wR*(*F*^2^) = 0.105	Δρ_max_ = 0.22 e Å^−^^3^
*S* = 1.03	Δρ_min_ = −0.18 e Å^−^^3^
2354 reflections	Extinction correction: *SHELXL2014/7* (Sheldrick, 2015), FC^*^=KFC[1+0.001XFC^2^Λ^3^/SIN(2Θ)]^-1/4^
158 parameters	Extinction coefficient: 0.0107 (9)
0 restraints	Absolute structure: see text
Hydrogen site location: mixed	

### Special details

**Table:**

Geometry. Bond distances, angles *etc*. have been calculated using the rounded fractional coordinates. All su's are estimated from the variances of the (full) variance-covariance matrix. The cell e.s.d.'s are taken into account in the estimation of distances, angles and torsion angles
Refinement. Refinement on *F*^2^ for ALL reflections except those flagged by the user for potential systematic errors. Weighted *R*-factors *wR* and all goodnesses of fit *S* are based on *F*^2^, conventional *R*-factors *R* are based on *F*, with *F* set to zero for negative *F*^2^. The observed criterion of *F*^2^ > σ(*F*^2^) is used only for calculating -*R*-factor-obs *etc*. and is not relevant to the choice of reflections for refinement. *R*-factors based on *F*^2^ are statistically about twice as large as those based on *F*, and *R*-factors based on ALL data will be even larger.

### Fractional atomic coordinates and isotropic or equivalent isotropic displacement parameters (Å^2^)

**Table:**

	*x*	*y*	*z*	*U*_iso_*/*U*_eq_	
O1	0.76749 (6)	0.89601 (14)	0.38299 (7)	0.0445 (3)	
N1	0.85894 (7)	0.5703 (2)	0.53117 (9)	0.0478 (3)	
C1	0.75159 (7)	0.65596 (19)	0.36005 (9)	0.0359 (3)	
C2	0.83327 (6)	0.5226 (2)	0.40885 (9)	0.0370 (3)	
C3	0.71413 (6)	0.60757 (19)	0.23561 (9)	0.0347 (3)	
C4	0.67124 (8)	0.4020 (2)	0.20194 (10)	0.0438 (3)	
C5	0.63480 (9)	0.3576 (2)	0.08879 (12)	0.0514 (4)	
C6	0.64086 (8)	0.5178 (3)	0.00766 (10)	0.0527 (4)	
C7	0.68404 (8)	0.7206 (3)	0.04003 (10)	0.0516 (4)	
C8	0.72072 (7)	0.7658 (2)	0.15340 (10)	0.0427 (3)	
C9	0.89766 (6)	0.57714 (19)	0.34642 (9)	0.0352 (3)	
C10	0.91137 (8)	0.4216 (2)	0.26755 (11)	0.0458 (4)	
C11	0.96758 (8)	0.4696 (3)	0.20612 (11)	0.0552 (4)	
C12	1.01117 (8)	0.6743 (3)	0.22244 (11)	0.0521 (4)	
C13	0.99884 (8)	0.8302 (2)	0.30109 (12)	0.0517 (4)	
C14	0.94275 (8)	0.7827 (2)	0.36305 (11)	0.0454 (4)	
H1	0.71190	0.60500	0.40080	0.0430*	
H2	0.82070	0.35640	0.39920	0.0440*	
H3	0.66700	0.29320	0.25600	0.0530*	
H4	0.60610	0.21950	0.06720	0.0620*	
H5	0.61590	0.48860	−0.06840	0.0630*	
H6	0.68870	0.82810	−0.01440	0.0620*	
H7	0.74990	0.90330	0.17440	0.0510*	
H8	0.88230	0.28220	0.25550	0.0550*	
H9	0.97580	0.36260	0.15350	0.0660*	
H10	1.04860	0.70720	0.18080	0.0630*	
H11	1.02840	0.96890	0.31290	0.0620*	
H12	0.93530	0.88950	0.41620	0.0540*	
H13	0.7225 (12)	0.959 (3)	0.4061 (15)	0.077 (5)*	
H14	0.8637 (12)	0.732 (4)	0.5400 (16)	0.085 (6)*	
H15	0.9080 (12)	0.508 (3)	0.5611 (15)	0.074 (5)*	

### Atomic displacement parameters (Å^2^)

**Table:**

	*U*^11^	*U*^22^	*U*^33^	*U*^12^	*U*^13^	*U*^23^
O1	0.0495 (5)	0.0365 (4)	0.0494 (5)	0.0033 (4)	0.0169 (4)	−0.0058 (3)
N1	0.0460 (6)	0.0600 (7)	0.0362 (5)	−0.0006 (5)	0.0089 (4)	0.0094 (5)
C1	0.0370 (5)	0.0355 (5)	0.0375 (5)	0.0012 (4)	0.0138 (4)	0.0019 (4)
C2	0.0378 (6)	0.0353 (6)	0.0381 (5)	0.0000 (4)	0.0106 (4)	0.0049 (4)
C3	0.0315 (5)	0.0362 (6)	0.0374 (5)	0.0048 (4)	0.0108 (4)	0.0026 (4)
C4	0.0496 (6)	0.0368 (6)	0.0456 (6)	−0.0001 (5)	0.0140 (5)	0.0032 (5)
C5	0.0540 (7)	0.0442 (7)	0.0536 (7)	−0.0054 (6)	0.0102 (6)	−0.0083 (6)
C6	0.0535 (7)	0.0617 (8)	0.0390 (6)	0.0018 (6)	0.0059 (5)	−0.0046 (6)
C7	0.0566 (7)	0.0565 (8)	0.0400 (6)	−0.0017 (6)	0.0104 (5)	0.0096 (6)
C8	0.0430 (6)	0.0419 (6)	0.0427 (6)	−0.0041 (5)	0.0108 (5)	0.0047 (5)
C9	0.0321 (5)	0.0346 (5)	0.0378 (5)	0.0039 (4)	0.0077 (4)	0.0051 (4)
C10	0.0417 (6)	0.0437 (7)	0.0525 (7)	−0.0027 (5)	0.0138 (5)	−0.0079 (5)
C11	0.0465 (7)	0.0714 (9)	0.0508 (7)	−0.0004 (6)	0.0186 (6)	−0.0137 (7)
C12	0.0375 (6)	0.0719 (9)	0.0495 (7)	0.0009 (6)	0.0162 (5)	0.0087 (6)
C13	0.0431 (6)	0.0468 (7)	0.0669 (8)	−0.0062 (5)	0.0176 (6)	0.0070 (6)
C14	0.0453 (6)	0.0382 (6)	0.0548 (7)	−0.0019 (5)	0.0171 (5)	−0.0028 (5)

### Geometric parameters (Å, º)

**Table:**

O1—C1	1.4213 (14)	C10—C11	1.386 (2)
N1—C2	1.4732 (15)	C11—C12	1.373 (2)
O1—H13	0.95 (2)	C12—C13	1.375 (2)
C1—C3	1.5138 (15)	C13—C14	1.388 (2)
N1—H15	0.88 (2)	C1—H1	0.9800
N1—H14	0.94 (2)	C2—H2	0.9800
C1—C2	1.5435 (16)	C4—H3	0.9300
C2—C9	1.5172 (15)	C5—H4	0.9300
C3—C8	1.3868 (16)	C6—H5	0.9300
C3—C4	1.3888 (16)	C7—H6	0.9300
C4—C5	1.3831 (19)	C8—H7	0.9300
C5—C6	1.382 (2)	C10—H8	0.9300
C6—C7	1.375 (2)	C11—H9	0.9300
C7—C8	1.3870 (17)	C12—H10	0.9300
C9—C14	1.3896 (16)	C13—H11	0.9300
C9—C10	1.3837 (17)	C14—H12	0.9300
			
C1—O1—H13	108.0 (11)	O1—C1—H1	108.00
O1—C1—C3	112.57 (9)	C2—C1—H1	108.00
C2—C1—C3	112.55 (9)	C3—C1—H1	108.00
H14—N1—H15	108.3 (17)	N1—C2—H2	107.00
O1—C1—C2	107.90 (9)	C1—C2—H2	107.00
C2—N1—H14	107.2 (12)	C9—C2—H2	107.00
C2—N1—H15	109.4 (12)	C3—C4—H3	120.00
N1—C2—C9	115.19 (9)	C5—C4—H3	120.00
C1—C2—C9	112.28 (9)	C4—C5—H4	120.00
N1—C2—C1	106.72 (9)	C6—C5—H4	120.00
C1—C3—C4	119.87 (10)	C5—C6—H5	120.00
C1—C3—C8	121.46 (10)	C7—C6—H5	120.00
C4—C3—C8	118.66 (10)	C6—C7—H6	120.00
C3—C4—C5	120.64 (11)	C8—C7—H6	120.00
C4—C5—C6	120.21 (12)	C3—C8—H7	120.00
C5—C6—C7	119.59 (12)	C7—C8—H7	120.00
C6—C7—C8	120.39 (13)	C9—C10—H8	119.00
C3—C8—C7	120.49 (12)	C11—C10—H8	119.00
C2—C9—C10	119.74 (10)	C10—C11—H9	120.00
C2—C9—C14	122.38 (10)	C12—C11—H9	120.00
C10—C9—C14	117.86 (11)	C11—C12—H10	120.00
C9—C10—C11	121.26 (12)	C13—C12—H10	120.00
C10—C11—C12	120.37 (13)	C12—C13—H11	120.00
C11—C12—C13	119.19 (13)	C14—C13—H11	120.00
C12—C13—C14	120.66 (12)	C9—C14—H12	120.00
C9—C14—C13	120.65 (11)	C13—C14—H12	120.00
			
O1—C1—C2—N1	59.72 (11)	C1—C3—C8—C7	−178.29 (12)
O1—C1—C2—C9	−67.39 (11)	C4—C3—C8—C7	0.98 (18)
C3—C1—C2—N1	−175.47 (9)	C3—C4—C5—C6	0.1 (2)
C3—C1—C2—C9	57.42 (12)	C4—C5—C6—C7	0.6 (2)
O1—C1—C3—C4	−159.70 (11)	C5—C6—C7—C8	−0.6 (2)
O1—C1—C3—C8	19.56 (15)	C6—C7—C8—C3	−0.2 (2)
C2—C1—C3—C4	78.10 (13)	C2—C9—C10—C11	177.69 (11)
C2—C1—C3—C8	−102.65 (12)	C14—C9—C10—C11	−0.64 (18)
N1—C2—C9—C10	136.09 (11)	C2—C9—C14—C13	−177.49 (11)
N1—C2—C9—C14	−45.66 (15)	C10—C9—C14—C13	0.80 (18)
C1—C2—C9—C10	−101.48 (12)	C9—C10—C11—C12	0.0 (2)
C1—C2—C9—C14	76.77 (13)	C10—C11—C12—C13	0.5 (2)
C1—C3—C4—C5	178.33 (12)	C11—C12—C13—C14	−0.4 (2)
C8—C3—C4—C5	−0.94 (19)	C12—C13—C14—C9	−0.3 (2)

### Hydrogen-bond geometry (Å, º)

*CgA* and *CgB* are the centroids of rings C3–C8 and C9–C14, respectively.

**Table:**

*D*—H···*A*	*D*—H	H···*A*	*D*···*A*	*D*—H···*A*
O1—H13···N1^i^	0.95 (2)	1.86 (2)	2.7977 (16)	173.1 (16)
N1—H15···*CgB*^ii^	0.88 (2)	2.670 (19)	3.5125 (14)	160.3 (15)
C12—H10···*CgA*^iii^	0.93	2.80	3.6780 (17)	158

Symmetry codes: (i) −*x*+3/2, *y*+1/2, −*z*+1; (ii) −*x*+2, −*y*+1, −*z*+1; (iii) *x*+1/2, −*y*+3/2, *z*.

## References

[bb1] Bari, A., Al-Obaid, A. M. & Ng, S. W. (2012). *Acta Cryst.* E**68**, o491. 10.1107/S1600536812002000 PMC3275237 22347093

[bb2] Enraf–Nonius (1989). *CAD-4 Software*. Enraf–Nonius, Delft, The Netherlands.

[bb3] Farrugia, L. J. (2012). *J. Appl. Cryst.* **45**, 849–854.

[bb4] Harms, K. & Wocadlo, S. (1995). *XCAD4*. University of Marburg, Germany.

[bb5] Imai, Y., Kawaguchi, K., Matsuno, H., Sato, T., Kuroda, R. & Matsubara, Y. (2008). *Tetrahedron*, **64**, 4585–4589.

[bb6] Kinbara, K., Kobayashi, Y. & Saigo, K. (1998). *J. Chem. Soc. Perkin Trans. 2*, pp. 1767–1776.

[bb7] Macrae, C. F., Bruno, I. J., Chisholm, J. A., Edgington, P. R., McCabe, P., Pidcock, E., Rodriguez-Monge, L., Taylor, R., van de Streek, J. & Wood, P. A. (2008). *J. Appl. Cryst.* **41**, 466–470.

[bb8] North, A. C. T., Phillips, D. C. & Mathews, F. S. (1968). *Acta Cryst.* A**24**, 351–359.

[bb9] Read, J. & Steele, C. C. (1927). *J. Chem. Soc.* pp. 910–918.

[bb10] Sheldrick, G. M. (2008). *Acta Cryst.* A**64**, 112–122. 10.1107/S0108767307043930 18156677

[bb11] Sheldrick, G. M. (2015). *Acta Cryst.* C**71**, 3–8.

[bb12] Spek, A. L. (2009). *Acta Cryst.* D**65**, 148–155. 10.1107/S090744490804362X PMC2631630 19171970

[bb13] Westrip, S. P. (2010). *J. Appl. Cryst.* **43**, 920–925.

